# Opportunistic infections (OIs) present in HIV-seropositive patients: a study

**DOI:** 10.1186/1758-2652-13-S4-P191

**Published:** 2010-11-08

**Authors:** K Ayodhya Ramaiah, B Sudha Rani, R Anand, S Ramola, P Satish Babu, R Ramesh Babu

**Affiliations:** 1 BOSS & CIPCA (CBO), Tirupati, India

## Background

HIV doesn't kill directly but it weakens the body's ability to fight disease. Infections, which are rarely seen in those with normal immune systems, are deadly to those with HIV. People with HIV can get many infections (opportunistic infections or OIs). Many of these illnesses are very serious, and they need to be treated. Some can be prevented.

## Purpose of the study

To study the spectrum of OIs occurring during HIV infection among seropositive patients.

## Methods

BOSS & CIPCA, a charitable non-Govt. voluntary community based organization, having 475 doctors and 8900 blood donors as members working on HIV/AIDS since 1987 has conducted 3 years (from January 2006 to December 2009) retrospective controlled study on the spectrum of OIs occurring during HIV infection among 10,500 seropositive patients attending VCTC and 50 bedded community care center run by BOSS & CIPCA. Their medical records were retrieved and scrutinized and the following data were extracted:

(1) Age, sex, race, occupation and marital status.

(2) Clinical manifestations with bacterial, viral, fungal, protozoal and other relevant neoplasms.

(3) Laboratory surrogate markers — routine haemogram, routine biochemical analysis, bacteriological, fungal examinations and serology.

(4) Radiological studies including ultrasound and CT scan where applicable.

## Summary of results

Out of the 10,500 seropositive patients only 3,703 have opportunistic infections. The most common bacterial infections were TB 38% (1407) (pulmonary and extra-pulmonary) and atypical pneumonia 12% (445) (streptococcal, staphylococcal, pneumoccocal and H. influenzae). In the protozoal group, malaria, helminthiasis and leishmaniasis 23% (852) were common. The common fungal infections were candidiasis, cryptococcosis and dermatophytosis 10% (370). Amongst the viral infections, Herpes zoster 8% (296) was most dominant followed by 9% (333) Hepatitis B and Herpes simplex infections

## Conclusions

TB was confirmed the most prevalent OI in the country. Differential diagnosis of these opportunistic infections may be useful in the early diagnosis of HIV infections in the various health facilities in the country. The study also highlights the regular availability of effective drugs for the treatment of the OIs and its preventive measures.
